# Targeting Oxidative Stress: The Potential of Vitamin C in Protecting against Liver Damage after Electron Beam Therapy

**DOI:** 10.3390/biomedicines12102195

**Published:** 2024-09-26

**Authors:** Grigory Demyashkin, Mikhail Parshenkov, Sergey Koryakin, Polina Skovorodko, Vladimir Shchekin, Vladislav Yakimenko, Zhanna Uruskhanova, Dali Ugurchieva, Ekaterina Pugacheva, Sergey Ivanov, Petr Shegay, Andrey Kaprin

**Affiliations:** 1 Department of Digital Oncomorphology, National Medical Research Centre of Radiology, 2nd Botkinsky Pass., 3, 125284 Moscow, Russia; korsernic@mail.ru (S.K.); dr.shchekin@mail.ru (V.S.); oncourolog@gmail.com (S.I.); dr.shegai@mail.ru (P.S.); kaprin@mail.ru (A.K.); 2 Laboratory of Histology and Immunohistochemistry, Institute of Translational Medicine and Biotechnology, I.M. Sechenov First Moscow State Medical University (Sechenov University), Trubetskaya st., 8/2, 119048 Moscow, Russia; misjakj@gmail.com (M.P.); skovorodko.polina2345@gmail.com (P.S.); keen2000@mail.ru (V.Y.); jey.149@yandex.ru (Z.U.); daliyagurchieva@gmail.com (D.U.); rouzella@mail.ru (E.P.); 3 Research and Educational Resource Center for Immunophenotyping, Digital Spatial Profiling and Ultrastructural Analysis Innovative Technologies, Peoples’ Friendship University of Russia (RUDN University), Miklukho-Maklaya st., 6, 117198 Moscow, Russia; 4 Department of Urology and Operative Nephrology, Peoples’ Friendship University of Russia (RUDN University), Miklukho-Maklaya str. 6, 117198 Moscow, Russia

**Keywords:** RT-PCR, radiation-induced liver disease, electron, apoptosis, Ki-67

## Abstract

*Background*: Radiation-induced liver disease (RILD) is a severe complication arising from radiotherapy, particularly when treating abdominal malignancies such as hepatocellular carcinoma. The liver’s critical role in systemic metabolism and its proximity to other abdominal organs make it highly susceptible to radiation-induced damage. This vulnerability significantly limits the maximum safe therapeutic dose of radiation, thereby constraining the overall efficacy of radiotherapy. Among the various modalities, electron beam therapy has gained attention due to its ability to precisely target tumors while minimizing exposure to surrounding healthy tissues. However, despite its advantages, the long-term impacts of electron beam exposure on liver tissue remain inadequately understood, particularly concerning chronic injury and fibrosis driven by sustained oxidative stress. *Objectives*: to investigate the molecular and cellular mechanisms underlying the radioprotective effects of vitamin C in a model of radiation-induced liver disease. *Methods:* Male Wistar rats (n = 120) were randomly assigned to four groups: control, fractionated local electron irradiation (30 Gy), pre-treatment with vitamin C before irradiation, and vitamin C alone. The study evaluated the effects of electron beam radiation and vitamin C on liver tissue through a comprehensive approach, including biochemical analysis of serum enzymes (ALT, AST, ALP, and bilirubin), cytokine levels (IL-1β, IL-6, IL-10, and TNF-α), and oxidative stress markers (MDA and SOD). Histological and morphometric analyses were conducted on liver tissue samples collected at 7, 30, 60, and 90 days, which involved standard staining techniques and advanced imaging, including light and electron microscopy. Gene expression of Bax, Bcl-2, and caspase-3 was analyzed using real-time PCR. *Results:* The present study demonstrated that fractional local electron irradiation led to significant reductions in body weight and liver mass, as well as marked increases in biochemical markers of liver damage (ALT, AST, ALP, and bilirubin), inflammatory cytokines (IL-1β, IL-6, and TNF-α), and oxidative stress markers (MDA) in the irradiated group. These changes were accompanied by substantial histopathological alterations, including hepatocyte degeneration, fibrosis, and disrupted microvascular circulation. Pre-treatment with vitamin C partially mitigated these effects, reducing the severity of the liver damage, oxidative stress, and inflammation, and preserving a more favorable balance between hepatocyte proliferation and apoptosis. Overall, the results highlight the potential protective role of vitamin C in reducing radiation-induced liver injury, although the long-term benefits require further investigation. *Conclusions*: The present study highlights vitamin C’s potential as a radioprotective agent against electron beam-induced liver damage. It effectively reduced oxidative stress, apoptosis, and inflammation, particularly in preventing the progression of radiation-induced liver fibrosis. These findings suggest that vitamin C could enhance radiotherapy outcomes by minimizing liver damage, warranting further exploration into its broader clinical applications.

## 1. Introduction

Radiation-induced liver disease (RILD) is a critical and often life-threatening complication arising from radiotherapy, especially in the treatment of abdominal malignancies [1,2]. As the liver plays a vital role in systemic metabolism and is anatomically close to other abdominal organs, it is frequently exposed to incidental radiation during therapeutic interventions, making it particularly susceptible to radiation-induced damage. This condition significantly constrains the maximum safe therapeutic dose of radiation, limiting the overall efficacy of radiotherapy protocols [3]. With the global incidence of hepatic malignancies continuing to rise, a comprehensive understanding of the differential impacts of various radiation modalities on liver tissue is crucial for optimizing radiotherapeutic strategies and reducing the risk of RILD.

Electron beam therapy has emerged as a pivotal modality in modern oncology due to its distinct physical properties, which offer substantial advantages over traditional X-ray and gamma radiation. Unlike photons, electrons have a limited penetration depth in tissue, enabling more precise tumor targeting while minimizing exposure to surrounding healthy tissues [4]. This characteristic makes electron beam therapy particularly effective for treating superficial tumors and has facilitated its widespread adoption in intra-operative electron radiation therapy (IOERT). IOERT exemplifies the integration of advanced surgical and radiotherapeutic techniques, allowing for the precise delivery of high radiation doses during surgery, thereby enhancing tumor control while sparing adjacent normal tissues [5,6]. Recent innovations in ultra-high dose rate electron beams, such as “FLASH-RT,” have further highlighted the potential of this modality, demonstrating a significant reduction in normal tissue toxicity without compromising anti-tumor efficacy in preclinical models [7,8].

Moreover, due to the reduced mass of electrons compared to photons, electron beam therapy is associated with a lower risk of inflicting direct DNA damage and generating free radicals in healthy liver tissues [9]. This presents electron beam therapy as a potentially safer alternative for patients with hepatic malignancies. However, the molecular and morphological mechanisms underlying electron-induced liver injury remain inadequately understood, as most existing research has focused on the acute effects of X-ray and gamma radiation, with limited exploration of the long-term impacts of electron beam exposure on liver tissue [10,11,12]. Given the liver’s susceptibility to chronic injury and fibrosis under sustained oxidative stress, there is an urgent need for comprehensive studies to fully elucidate these long-term effects to optimize the application of electron beam therapy and maximize its therapeutic potential.

RILD is primarily driven by oxidative stress, which activates complex molecular pathways leading to significant hepatic injury. Following radiation exposure, the overproduction of reactive oxygen species (ROS) results in mitochondrial dysfunction and mitochondrial DNA (mtDNA) damage, sensitizing hepatocytes and activating non-parenchymal cells such as Kupffer cells (KCs) and hepatic stellate cells (HSCs) [1,13,14]. These activated cells further exacerbate liver damage by releasing pro-inflammatory cytokines, including TNF-α, IL-1, and IL-6, which amplify the inflammatory response [15,16]. Additionally, critical signaling pathways involving NF-κB and Nrf2 play pivotal roles in modulating the liver’s response to radiation-induced damage. NF-κB serves as a central mediator of inflammation by promoting the expression of pro-inflammatory genes, while Nrf2 regulates antioxidant defenses, providing protection against ROS-induced damage [17,18]. As oxidative stress intensifies, key enzymes such as superoxide dismutase (SOD) attempt to neutralize superoxide radicals, while malondialdehyde (MDA) serves as a marker of lipid peroxidation and oxidative damage within liver tissues [18,19]. Despite these protective mechanisms, prolonged oxidative stress often overwhelms them, leading to sustained activation of HSCs and increased levels of the fibrogenic cytokine TGF-β, which stimulates the synthesis of extracellular matrix components like collagen, promoting fibrosis and long-term liver damage [20]. The chronic nature of radiation injury, tipping the balance toward persistent fibrogenesis and apoptosis, underscores the complexity of RILD. This intricate interplay of oxidative stress, inflammation, and signaling pathways necessitates further research to elucidate the specific molecular mechanisms involved in electron beam-induced hepatic injury.

Based on the limitations of previous approaches, our study focused on ascorbic acid (vitamin C) as a potential radioprotective agent for the liver. Known for its potent antioxidant properties, vitamin C neutralizes free radicals by donating electrons [21]. Its structure, characterized by multiple hydroxyl groups, facilitates redox reactions, including the reduction of metal ions like iron and copper, which are crucial for various enzymatic processes [22]. Moreover, vitamin C aids in regenerating other antioxidants such as vitamin E and influences gene expression by modulating epigenetic regulators like TET enzymes [23]. By mitigating oxidative stress and bolstering the liver’s endogenous antioxidant defenses, ascorbic acid holds significant promise in protecting against radiation-induced liver fibrosis and other damage.

Thus, this study addressed a critical gap in understanding the effects of electron beam radiation on the liver, particularly in relation to the potential radioprotective properties of vitamin C and explored the intricate roles of antioxidant and anti-inflammatory pathways in mitigating long-term radiation-induced hepatic damage.

### Research Objective

*The aim of the study* was to conduct a comprehensive analysis of the molecular and cellular mechanisms underlying the radioprotective effects of vitamin C in a model of radiation-induced liver disease. Specifically, the study focused on the impact of electron beam radiation on hepatocyte viability and inflammatory response markers. By exploring the role of vitamin C in modulating oxidative stress and the activation of signaling pathways related to fibrogenesis, this research aimed to uncover new therapeutic strategies for mitigating long-term radiation-induced liver damage.

## 2. Material and Methods

### 2.1. Experimental Animals

Wistar rats (male; 8–9 weeks; 220 ± 20 g; n = 120) were selected as the experimental model. The ambient temperature was maintained at 22–23 °C, with a 12 h light/dark cycle (12 L:12 D) to replicate natural circadian rhythms, which are critical for maintaining consistent metabolic and behavioral patterns The humidity was regulated to be between 40 and 60%, and the rats had free access to standard laboratory chow and water. The animals were housed individually within plastic cages [40 cm width (W) × 30 cm length (L) × 25 cm height (H)], lined with absorbent material (rice husk) to provide suitable nesting material and to reduce the stress associated with solitary confinement, which could influence their behavior and physiological responses.

Prior to the commencement of the study, blood samples were collected from all animals to assess the stability and normality of their physiological parameters. Animals displaying any deviations from the established normal ranges were excluded from the study to ensure the reliability of the experimental outcomes. Subsequently, the remaining animals were randomly assigned to their respective groups, a process intended to eliminate bias and ensure that the results obtained across the groups were not influenced by pre-existing physiological differences.

### 2.2. Experimental Design

**Figure 1:**
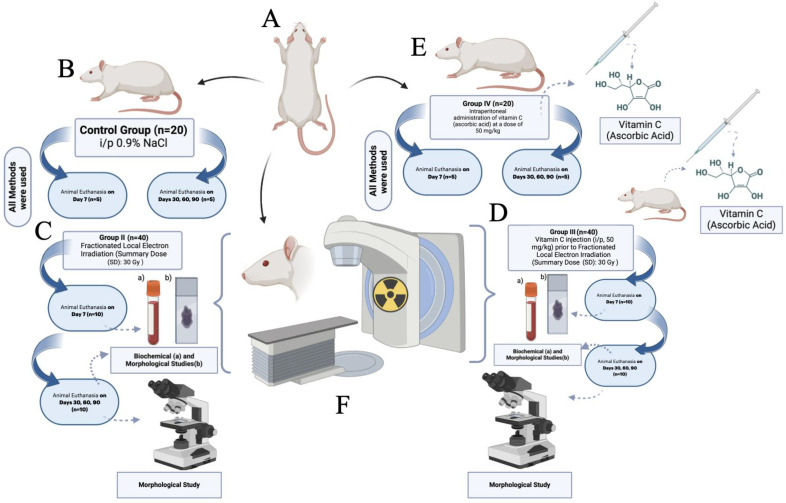
Design of the experiment. Special annotations: (**A**)—random allocation of animals into groups (following preliminary assessments); (**B**)—control group (animals received 0.9% saline i/p injection); (**C**)—Group II: animals received a Summary Fractional Dose of 30 Gy; (**D**)—Group III: prior to irradiation with a Summary Fractional Dose of 30 Gray, animals received vitamin C (ascorbic acid, 50 mg/kg, i/p injection); (E)—Group IV: animals received vitamin C i/p injections (ascorbic acid, 50 mg/kg); (**F**)—irradiation of the animals was performed using a specialized device (NOVAC-11, S.I.T. Sordina IORT Technologies S.P.A., Vicenza, Italy) according to the study design and methods.

The rats (n = 120) were divided into groups according to the design of the experiment (Figure 1):

- Group I (n = 20)—Control (intact);
- Group II (n = 40)—Fractionated local electron irradiation (Summary Dose (SD): 30 Gy);
- Group III (n = 40)—Intraperitoneal administration of vitamin C (ascorbic acid) at a dose of 50 mg/kg was performed 60 min prior to fractionated local electron irradiation (Summary Dose (SD): 30 Gy);
- Group IV (n = 20)—Intraperitoneal administration of vitamin C (ascorbic acid) at a dose of 50 mg/kg;

Animals from all groups were euthanized using high doses of anesthetics (ketamine (Supriya Lifescience, Ratnagiri, India) at a dose of 50 mg/kg intramuscularly and xylazine (Alivira Animal Health, Hyderabad, India) at a dose of 5 mg/kg intraperitoneally) on days 7, 30, 60, and 90 of the experiment. At all experiment time points, liver tissue was collected for biochemical analysis, preparation of homogenates, morphological evaluation, and PCR analysis.

### 2.3. RILD Model

The upper abdominal segments of the animals in Groups II, III, and IV were subjected to localized electron irradiation, which was carried out in fractions of 5 Gy (a total of 6 procedures with an interval of 24 h in between), resulting in a total irradiation dose of 30 Gy (dose rate: 1 Gy/min; energy: 10 MeV; frequency: 9 Hz; field size: Ø 50 mm). To simulate RILD, the linear electron accelerator “NOVAC-11” (Sordina IORT Technologies S.p.A., Italy) situated in the Radiological Department of the experimental facility at the A.F. Tsyba MRRC (Obninsk, Russia) was employed.

Prior to irradiation, rats in the experimental groups were sedated with an intramuscular injection of ketamine (50 mg/kg) and xylazine (5 mg/kg). The anesthetized animals were positioned prone on an examination table, with their limbs secured using specialized restraint devices. To protect non-targeted organs, such as the heart and lungs, shielding was applied outside the irradiation zone. The irradiation tube was positioned perpendicularly to the liver, with its end no more than two millimeters from the skin for maximum precision.

The physical appearance and weight of each animal, both absolute (in grams) and relative to body weight (in %), were meticulously recorded and analyzed. Additionally, the size and condition of the liver parenchyma were carefully evaluated upon dissection. Throughout the study, animal welfare remained a top priority, with all necessary precautions taken to minimize discomfort and stress, ensuring that the procedures were conducted humanely and ethically.

### 2.4. Vitamin C

Vitamin C (ascorbic acid) (Pharmstandard, Ufa, Russia) was administered intraperitoneally, at a dose of 50 mg/kg, 60 min prior to each session of electron beam irradiation, allowing sufficient time for absorption and the onset of its protective effects. The dose of ascorbic acid (50 mg/kg) was determined in preliminary studies under experimental conditions where several doses of vitamin C (30 mg/kg, 50 mg/kg, 100 mg/kg, and 200 mg/kg) were evaluated. The selected dose was found to provide an optimal balance between efficacy and safety, demonstrating a significant protective effect and no adverse reactions.

### 2.5. Biochemical Analysis of Blood Serum

The levels of alanine aminotransferase (ALT), aspartate aminotransferase (AST), alkaline phosphatase (ALP), and total bilirubin in blood samples collected from the animals were quantified using a Hitachi 7080 biochemical analyzer (Hitachi, Tokyo, Japan).

The levels of cytokines IL-1β, IL-6 (Bender MedSystems, Vienna, Austria), IL-10 (Abcam, Branford, CT, USA), and TNF-α (Assaypro, St. Charles, MO, USA) in the serum of the animals were measured. These measurements were conducted using commercial ELISA kits (Cloud-Clone Corp., Houston, TX, USA) following the manufacturer’s instructions.

### 2.6. Assessment of Oxidative Stress Markers

Liver tissues (200 mg) were homogenized and centrifuged for 5 min at 1000× *g* in an ice-cold solution to obtain a 10% homogenate to evaluate the oxidant/antioxidant ratio. The homogenate was centrifuged, and the supernatant was collected for further analysis. The levels of malondialdehyde (MDA) and superoxide dismutase (SOD) were analyzed according to the requirements of the ELISA kit (Lifespan Biosciences, Shirley, MA, USA).

### 2.7. Histological, Morphometric, and Histochemical Analyses

The liver samples were fixed in buffered formalin, processed using an automated system, and embedded in paraffin blocks. Serial sections, each 3 μm thick, were prepared from these blocks, followed by deparaffinization, dehydration, and staining with hematoxylin and eosin. The resulting histological slides were examined under a Leica DM2000 microscope (Leica Microsystems, Wetzlar, Germany), and microphotographs were taken for detailed analysis. The degree of liver damage was assessed using established histological criteria, as outlined by Knodell et al., with a numerical scoring system specifically designed for evaluating histological activity in asymptomatic chronic active hepatitis [24].

The Image J software (Ver. 1.52u) was utilized to calculate the following parameters: volumetric density of hepatocytes, average hepatocyte diameter (in µm), average diameter of the central vein (in µm), and the number of Kupffer cells per square centimeter. Morphometric assessments were conducted in 10 randomly selected fields of view at ×400 magnification, across 5 random sections per sample, using the Leica Application Suite (LAS) version 4.9.0 image analysis software.

### 2.8. Immunohistochemistry (IHC) Staining

For immunohistochemical (IHC) analysis of hepatocyte life cycle markers, primary monoclonal antibodies targeting Ki-67 (clone MM1; Thermo Fisher Scientific, Waltham, MA, USA) and caspase-3 (clone 74T2; Thermo Fisher Scientific, USA) were employed. To visualize secondary antibodies, we utilized the HiDef Detection™ HRP Polymer system (Cell Marque, Rocklin, CA, USA), which includes anti-rabbit/mouse IgG conjugated with horseradish peroxidase (HRP) and a DAB substrate kit. Cell nuclei were counterstained with Mayer’s hematoxylin solution. The number of immunopositive cells was quantified in 10 randomly selected fields of view at ×400 magnification, expressed as a percentage.

### 2.9. Real-Time PCR

To analyze the expression of the Bax, Bcl-2, and caspase-3 genes in liver tissues following electron beam irradiation and ascorbic acid administration, real-time PCR was employed. Total RNA was extracted from liver samples using the TRIzol reagent (Invitrogen), and cDNA synthesis was performed using RNA transcription kits from Promega Corporation (Madison, WI, USA). The specific primer pairs for Bax, Bcl-2, and caspase-3 genes are listed in Table 1.

Real-time PCR was conducted with 10 samples from each group, and each sample was run in triplicate to ensure accuracy and reproducibility. DEPC-treated water, 2X Tap, PCR Mix, and DNA markers from Promega Corporation were used in the reactions. The amplification conditions were optimized according to the primer specifications and experimental requirements.

### 2.10. Additional Information

The study’s primary focus was on the initial (7 days) and long-term changes (90 days) based on a review of the literature and our own preliminary experiments in related models and organs. At the same time, the study methods were thoroughly applied across all experimental periods—7, 30, 60, and 90 days.

### 2.11. Statistical Analysis

The data obtained from the calculations were processed using SPSS 12 for Windows (IBM Analytics, Armonk, NY, USA). The results are expressed as the mean ± standard deviation (SD). The Shapiro–Wilk test was used to assess the normality of the data distribution. For comparisons between study groups with non-normal distributions, the Kruskal–Wallis test followed by Dunn’s post hoc test was applied. Multiple comparisons were performed using the Mann–Whitney U test. A *p*-value ≤ 0.05 was considered statistically significant.

## 3. Results

### 3.1. Changes in Animal Weight and Liver Mass

At the beginning of the study, the initial body weight of all the animals was approximately 200 ± 20 g, with no significant differences observed between the groups. Animals whose weights deviated significantly from this range were replaced with others to ensure consistency across the experimental groups. Over time, the body weight increased in all groups, although the degree of increase varied according to the experimental conditions.

**Figure 2:**
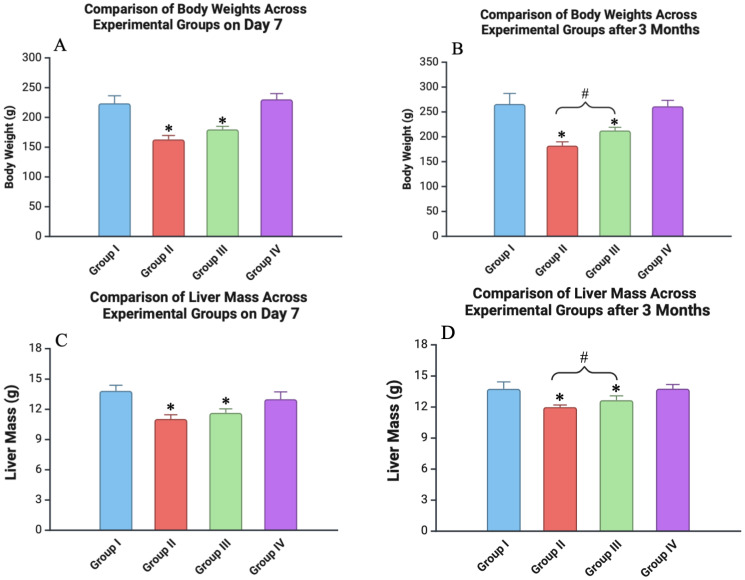
Comparison of body weight and liver mass across experimental groups at different time points: (**A**) body weight of animals on day 7; (**B**) body weight of animals after 3 months; (**C**) liver mass of animals on day 7; (**D**) liver mass of animals after 3 months. Experimental groups are numbered according to the study design. All data are presented as mean ± SD. Statistically significant differences are indicated by symbols: *—comparison with control group (*p* < 0.05); #—comparison between Groups II and III (*p* < 0.05).

After 7 days, Group II (irradiation only) exhibited a significant reduction in body weight, with an average decrease of 19% relative to the control group (*p* < 0.05). Animals in Group III (irradiation + ascorbic acid) also showed a decrease in body weight relative to the control, although this reduction was slightly less pronounced, averaging 14% (*p* < 0.05) (Figure 2).

During the subsequent time points at 30 and 60 days, there was a trend of gradually increasing body masses across all experimental groups.

After 3 months, the body weight of the animals in the control group had increased by an average of 21.8%. A similar trend was observed in Group IV (Vit. C only), which did not differ significantly from the control animals (*p* > 0.05). The weight of the animals in Groups II and III increased after 90 days, but it was still less than the control values (22.7% and 16% lower; *p* < 0.05) (Figure 2).

The liver mass was systematically measured across all experimental groups at each time point. In both the control group and the group receiving only ascorbic acid (Group IV), the liver mass remained relatively stable over the course of the study. Conversely, animals in the irradiation-only group (Group II) experienced a reduction in liver mass, with a decrease of 9.8% observed after 7 days compared to the control group (*p* < 0.05) (Figure 2). Although a slight recovery in liver mass was noted over the subsequent 3 months, including positive trends at the 1- and 2-month time points, the liver weight in this group remained significantly lower than that of the control group (*p* < 0.05). In the group pre-treated with ascorbic acid before irradiation (Group III), the decrease in liver weight was less severe, with an 8.1% reduction observed after 7 days compared to the control (*p* < 0.05). By the 3-month mark, an increase in liver weight was recorded in this group relative to the 7-day values. Nonetheless, the liver mass in the Vit. C + Irradiated group remained significantly lower than that of the control group (*p* < 0.05) (Figure 2).

### 3.2. Biochemical Analysis

**Figure 3:**
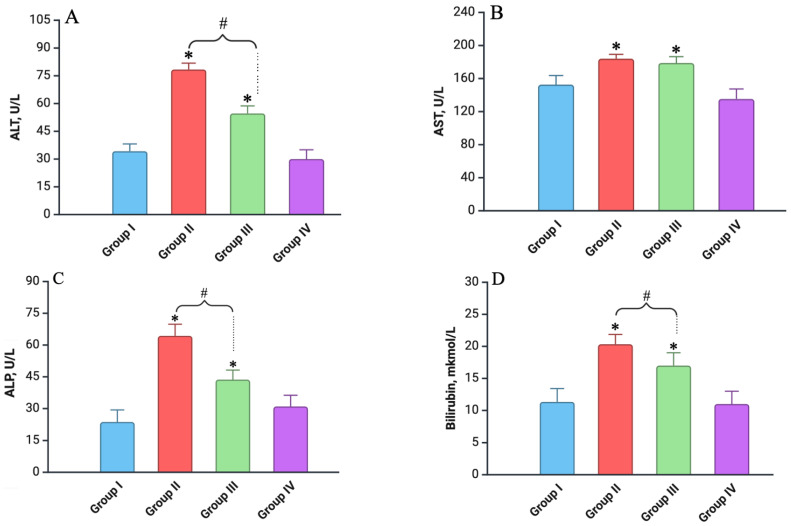
Biochemical analysis of blood serum (on day 7): (**A**) ALT (alanine aminotransferase) level; (**B**) AST (aspartate aminotransferase) level; (**C**) ALP (alkaline phosphatase) level; (**D**) total bilirubin level. Experimental groups are numbered according to the study design. All data are presented as mean ± SD. Statistically significant differences are indicated by symbols: *—comparison with control group (*p* < 0.05); #—comparison between Groups II and III (*p* < 0.05).

Biochemical markers of liver function were measured across all experimental groups at each time point. In the control group, as well as in the Vit. C-only group, the levels of ALT, AST, ALP, and total bilirubin remained stable over time. In contrast, the animals in Group II (irradiated only) exhibited a significant increase in these markers just 7 days after exposure, with ALT, AST, ALP, and total bilirubin levels rising by significant margins compared to the control group (*p* < 0.05) (Figure 3). This increase indicates acute liver damage and impaired hepatic function. However, during the following 30, 60, and 90 days, these values returned to baseline levels that were comparable to the control, reflecting a recovery process. In the group that received ascorbic acid before irradiation (Group III), the elevation in biochemical markers was less pronounced after 7 days compared to the irradiation-only group (*p* < 0.05). Like the other groups, the levels in Group III (Vit. C + Irradiated) also returned to baseline over the course of the 90-day study period, further suggesting a protective effect of vitamin C against radiation-induced liver damage (Figure 3).

**Figure 4:**
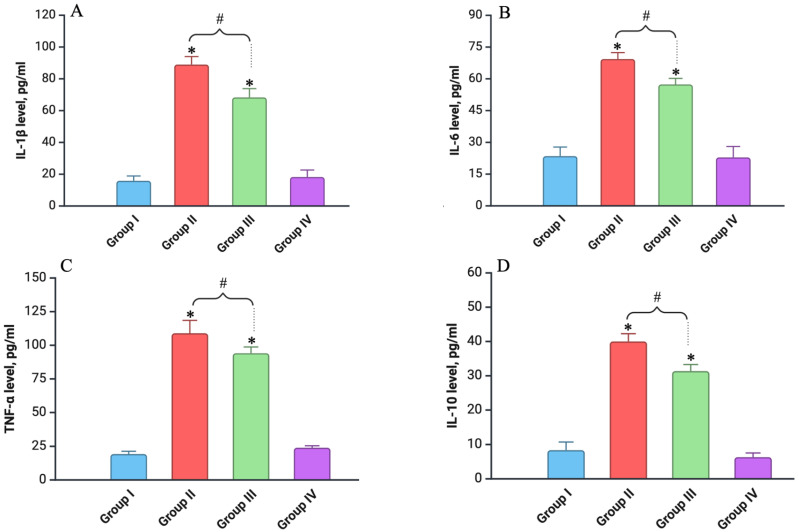
Levels of different cytokines in blood serum of experimental groups (on day 7): (**A**) level of IL-1β; (**B**) level of IL-6; (**C**) level of TNF-α; (**D**) level of IL-10. Experimental groups are numbered according to the study design. All data are presented as mean ± SD. Statistically significant differences are indicated by symbols: *—comparison with control group (*p* < 0.05); #—comparison between Groups II and III (*p* < 0.05).

One week after fractional local irradiation with electrons, there was a notable increase in inflammatory cytokine levels in Groups II and III compared to the control, with the extent of the increase depending on the irradiation condition (Figure 4).

In the irradiated-only group, the IL-1β levels exhibited a significant rise, increasing by 6.2 times compared to the control (*p* < 0.05), highlighting the strong inflammatory response induced by the irradiation. In Group III (Vit. C + Irradiated), where animals were pre-treated with vitamin C, the increase in IL-1β was attenuated to 5.1 times compared to the control (*p* < 0.05), suggesting a protective effect of vitamin C (Figure 4A). Similarly, the IL-6 levels were markedly elevated in the irradiation-only group, showing a 3.1-fold increase relative to the control (*p* < 0.05). However, in the vitamin C pre-treated group, the increase was reduced to 2.6 times (*p* < 0.05), further indicating the mitigating influence of vitamin C on the inflammatory response (Figure 4B). The TNF-α levels showed the most pronounced spike, with a 6.8-fold rise in the irradiation-only group (*p* < 0.05), underscoring the severity of the inflammatory reaction. The vitamin C pre-treated group, however, exhibited a slightly lower increase of 5.3 times (*p* < 0.05), reflecting the potential of vitamin C to attenuate TNF-α-mediated inflammation (Figure 4C). Moreover, the levels of the anti-inflammatory cytokine IL-10 also increased in both groups following irradiation. In the irradiated-only group, the IL-10 levels rose by 5.2 times compared to the control (*p* < 0.05), while in Group III (Vit. C + Irradiated), the increase was slightly less, at 4.7 times (*p* < 0.05), indicating that vitamin C may also modulate the anti-inflammatory response to some extent (Figure 4D).

On days 30, 60, and 90, the metrics of all the experimental groups were comparable to those of the control (*p* > 0.05).

### 3.3. Oxidative Stress Marker Analysis

**Figure 5:**
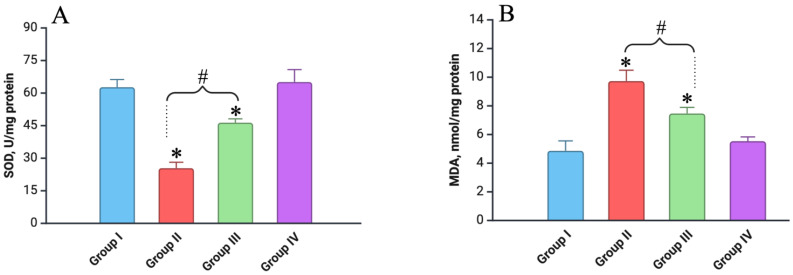
Levels of different markers of oxidative stress in liver homogenate of experimental groups (on day 7): (**A**) level of SOD; (**B**) level of MDA. Experimental groups are numbered according to the study design. All data are presented as mean ± SD. Statistically significant differences are indicated by symbols: *—comparison with control group (*p* < 0.05); #—comparison between Groups II and III (*p* < 0.05).

The assessment of oxidative stress markers in liver tissue homogenates a week after fractional electron irradiation at a total dose of 30 Gy revealed significant alterations in oxidative stress levels across the experimental groups compared to the control. The level of malondialdehyde (MDA), a marker of lipid peroxidation, showed a marked increase, while the activity of superoxide dismutase (SOD), a key antioxidant enzyme, was significantly reduced (Figure 5).

In the irradiated-only group, the MDA levels were found to increase by 2.4 times compared to the control (*p* < 0.05), highlighting substantial oxidative stress. Concurrently, the SOD activity decreased by 2.6 times relative to the control (*p* < 0.05), indicating a significant depletion of the antioxidant defenses. These results underscore the oxidative damage induced by electron irradiation. In contrast, Group III (Vit. C + Irradiated), which received vitamin C prior to irradiation, exhibited less pronounced changes. The MDA levels in this group increased by 1.6 times compared to the control (*p* < 0.05), while the reduction in SOD activity was limited to 1.4 times (*p* < 0.05). This suggests that vitamin C provided a partial protective effect against the oxidative stress induced by the irradiation. In the Vit. C-only group, both the MDA and SOD levels remained comparable to those of the control (*p* > 0.05), indicating no significant oxidative stress in the absence of irradiation.

On days 30, 60, and 90, the oxidative stress markers in all the experimental groups had returned to levels comparable to the control, suggesting a recovery over time.

### 3.4. Histological Analysis

In the control group, a normal histoarchitecture of the liver parenchyma was noted. The HAI score was 0 points. A similar morphological picture was present in the Vit. C-only group.

**Figure 6:**
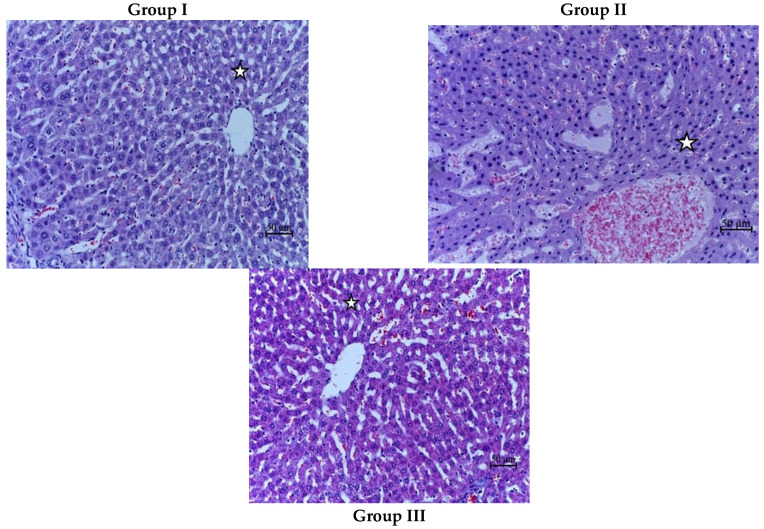
H&E stained liver fragments from the control (Group I), irradiated group (Group II), irradiated group with the introduction of ascorbic acid (Group III). Magn.: ×400; star—central vein; scale bar—50 µm.

In the group that received fractional local electron irradiation at a dose of 30 Gy, small cysts were observed after a week. There was also ballooning degeneration of most hepatocytes (cytoplasmic vacuolization and karyopyknosis), mainly in zone III; local atrophy of one-third to two-thirds of the hepatic lobules; moderate partial necrosis (less than 50% of the circumference of most portal tracts); and cellular inflammatory infiltration of one-third to two-thirds of the portal tracts (including mononuclear cells). There was no fibrosis (on average, 9 points on the HAI scale). In addition, sinusoidal dilatation and congestion, Kupffer cell hyperplasia, perisinusoidal hemorrhages, and hyperplasia of the bile duct wall were found (Figure 6). In terms of dynamics, we noted a decrease in the degree of severity of the detected pathomorphological changes. Three months after exposure to local electron irradiation at a total dose of 30 Gy, moderate proliferation of the fibrous component, atrophy of one-sixth of the hepatic lobules, and single mononuclear inflammatory cells were observed. In some places, hyperplasia of Kupffer cells, sinusoidal dilatation, and bile duct wall thickening were noted. These changes were identified as signs of radiation-induced liver fibrosis within the RILD framework.

The administration of vitamin C in Group III led to less pronounced liver damage in accordance with morphological criteria: the inflammatory infiltrate occupied less than one-third of the portal tracts and fibrosis was absent (average HAI score 4) (Figure 6), with subsequent improvement of the morphological picture in terms of dynamics. In the group that received pre-radiation administration of vitamin C, in the third month of the experiment, practically no proliferation of the fibrous component of the liver was detected, and focal atrophy of hepatocytes was observed only in a single sample. There were no inflammatory cells.

### 3.5. Morphometric Analysis

**Figure 7:**
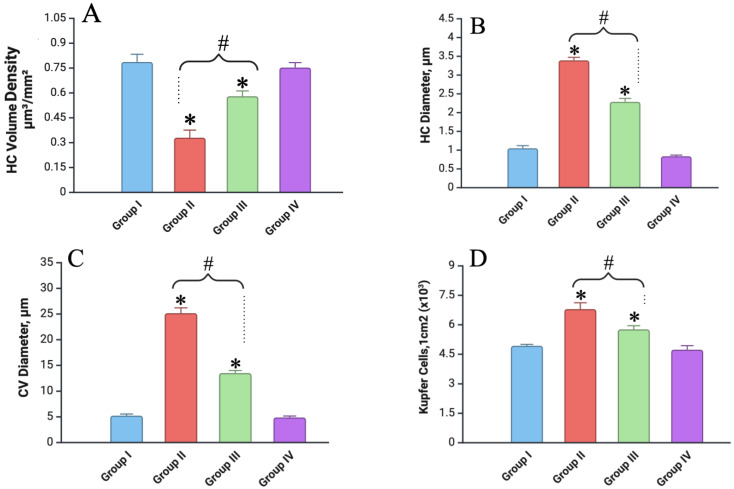
Liver studies of all experimental groups (on 7 days): (**A**) indicators of volumetric density of hepatocytes (HC) (in µm^3^;/mm^2^); (**B**) diameter of hepatocytes (in µm); (**C**) diameter of the central vein (CV, in µm); (**D**) number of Kupffer cells in 1 cm^2^ (×10^3^). Experimental groups are numbered according to the study design. All data are presented as mean ± SD. Statistically significant differences are indicated by symbols: *—comparison with control group (*p* < 0.05); #—comparison between Groups II and III (*p* < 0.05).

In the morphometric analysis of the liver a week after fractional local irradiation, sharp decreases in the volume density (a decrease in the number of hepatocytes per unit volume) and the diameter of hepatocytes were found, which were accompanied by an expansion of the diameter of the central vein (in 4.3 times) and hyperplasia of Kupffer cells compared to the control values (Figure 7). In terms of dynamics, we noted a decrease in the degree of severity of the detected morphometric defects.

At one week after fractional local irradiation, the group that received pre-irradiation administration of vitamin C had a less pronounced decrease in the volume density and diameter of hepatocytes and a slight increase in the diameter of the central vein (in 2.3 times) and the number of Kupffer cells compared to the control values. We discovered subsequent recovery of the morphometric parameters in terms of dynamics.

The morphometry of the Vit. C-only group was the same as that of the control (Figure 7).

### 3.6. Life Span of Hepatocytes

**Figure 8:**
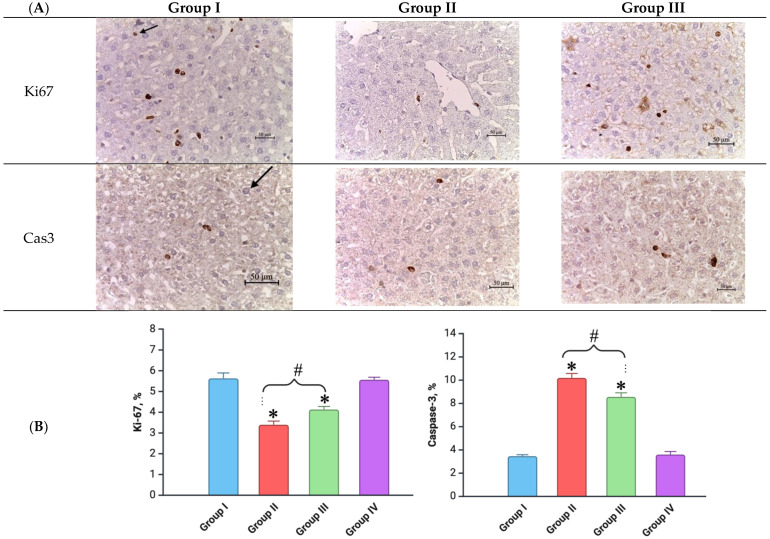
Livers of the control and experimental groups: (**A**) immunohistochemical reactions with antibodies to Ki-67 (top row) and caspase 3 (bottom row) (magnification: ×400); (**B**) proliferation–apoptosis ratio in hepatocytes according to the results of an immunohistochemical study (scale bar—50 µm). Experimental groups are numbered according to the study design. All data are presented as mean ± SD. Statistically significant differences are indicated by symbols: *—comparison with control group (*p* < 0.05); #—comparison between Groups II and III (*p* < 0.05).

An immunohistochemical (IHC) study was conducted on day 7 to evaluate the balance between cell proliferation and apoptosis in liver tissue across the different experimental groups. In the control group, a baseline level of hepatocyte proliferation was observed, with Ki-67-positive staining in approximately 5.7 ± 0.2% of cells, alongside apoptosis markers detected in 6.4 ± 0.3% of cells that were positively stained for caspase-3. Non-parenchymal cells, including fibroblasts and endothelial cells, also showed occasional staining, which is consistent with normal liver turnover and homeostasis (Figure 8). On day 90, the levels of Ki-67 and caspase-3 in all the experimental groups returned to levels comparable to the control, suggesting that any early disruptions in cell turnover had resolved over time.

However, in the group subjected to fractional electron irradiation at a cumulative dose of 30 Gy (irradiated-only group), there was a marked shift in the proliferation–apoptosis balance after one week. The proportion of Ki-67-positive hepatocytes decreased significantly to 2.6 ± 0.1% (*p* < 0.05), indicating reduced mitotic activity. Conversely, caspase-3 staining increased to 12.5 ± 0.6% (*p* < 0.05), reflecting a heightened rate of apoptosis among hepatocytes. These changes were accompanied by a slight increase in the proportion of Ki-67 and caspase-3-positive non-parenchymal cells, such as fibroblastic cells, sinusoidal endothelial cells, and those lining small- to medium-sized blood vessels (Figure 8). This suggests a broader impact of irradiation on both the parenchymal and stromal components of the liver.

Interestingly, the administration of vitamin C prior to fractional electron irradiation (Group III) mitigated these effects to a significant extent. In this group, the Ki-67-positive staining in hepatocytes was moderately higher at 3.3 ± 0.1% (*p* < 0.05) compared to the irradiation-only group, while the caspase-3 positivity was lower, at 10.3 ± 0.5% (*p* < 0.05) (Figure 8). These findings suggest that vitamin C helps to preserve the proliferative capacity of hepatocytes while reducing apoptosis, thereby maintaining a more favorable proliferation–apoptosis balance in the liver tissue. The staining pattern for non-parenchymal cells in this group was largely comparable to the control values, indicating minimal disruption to the liver’s stromal architecture.

### 3.7. RT-PCR Analysis

**Figure 9:**
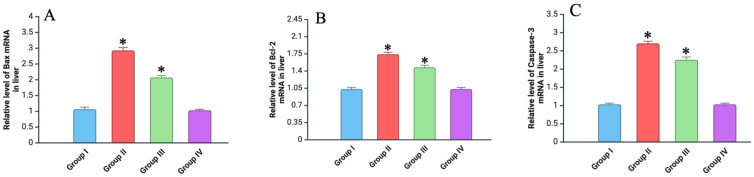
RT-PCR analysis of different markers (on day 7): (**A**) relative levels of Bax mRNA; (**B**) relative levels of Bcl-2 mRNA; (**C**) relative levels of caspase-3 mRNA. Experimental groups are numbered according to the study design. All data are presented as mean ± SD. Statistically significant differences are indicated by symbols: *—comparison with control group (*p* < 0.05).

To evaluate the effects of electron beam irradiation and vitamin C administration on the expression levels of the Bax, Bcl-2, and caspase-3 genes in liver tissues, real-time PCR was performed. The results (on day 7) demonstrated significant alterations in gene expression across the experimental groups, consistent with the expected apoptotic and anti-apoptotic responses. For example, in the second group, a substantial upregulation of Bax mRNA expression was observed compared to the control group, indicating an increase in pro-apoptotic signaling (Figure 9). Conversely, the expression of Bcl-2, an anti-apoptotic gene, was also elevated, albeit to a lesser extent. This resulted in an increased Bax/Bcl-2 ratio, suggesting a tilt towards apoptosis in the liver cells post-irradiation. In contrast, the third group exhibited a moderate response. While both Bax and Bcl-2 expression was still elevated compared to the control, the increase was significantly less pronounced than in the irradiation-only group. This was reflected in a more balanced Bax/Bcl-2 ratio, implying that vitamin C administration may help in reducing the extent of apoptosis triggered by irradiation.

Moreover, caspase-3, a crucial effector of apoptosis, showed a marked increase in its expression in the irradiated group (Figure 9). The elevation in caspase-3 mRNA was significantly higher than in the control, correlating with the observed increase in apoptotic activity. However, in the group treated with vitamin C, the rise in caspase-3 expression was less significant, reinforcing the potential protective role of vitamin C against radiation-induced apoptosis.

At the conclusion of the study (60 and 90 days), no statistically significant differences were observed between the study groups. However, on day 30, the values in Group II were slightly higher compared to those of the other groups.

## 4. Discussion

**Figure 10:**
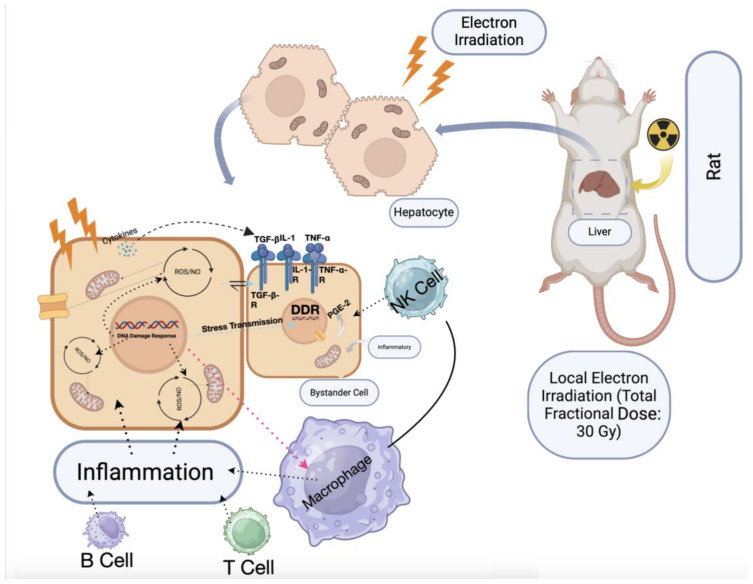
Alterations in gene expression pathways linked to inflammation due to the radiation-induced bystander effect. Alterations in gene expression involved in the bystander effect, such as MAPKs, NF-κB, iNOS, and COX-2 expression, play a significant role in promoting inflammation and oxidative stress in liver tissue following radiation exposure. These changes are driven by cytokines like IL-1, TGFβ, and TNFα, which activate signaling pathways leading to the upregulation of COX-2 and iNOS. This upregulation increases the production of pro-inflammatory molecules, contributing to tissue inflammation and damage in both irradiated and surrounding non-irradiated cells.

Radiation-induced liver disease (RILD) presents a significant challenge in the treatment of abdominal malignancies, particularly hepatocellular carcinoma, as it restricts the maximum safe therapeutic dose of radiation, thereby limiting the effectiveness of radiotherapy [30]. RILD is associated with a worsening prognosis and reduced quality of life, and can progress to liver cirrhosis or even liver failure [31]. Although high radiation doses (74 Gy and above) are considered most effective for treating hepatocellular carcinoma, even lower doses (30–35 Gy) have been associated with the onset of classical RILD in 5–10% of patients [32]. Administering doses above 60 Gy significantly increases mortality risk, with rates reaching up to 76%, largely due to the rapid onset of radiation-induced liver failure and damage to the normal liver parenchyma, often exacerbated by the bystander effect (Figure 10) [33]. These complications frequently lead to long-term effects such as parenchymal fibrosis and liver cirrhosis.

The literature reports that RILD manifests in 100% of animals subjected to even low doses of X-irradiation, with typical presentations including hepatocyte edema, hemorrhages, and sinusoidal congestion, which are observed at doses as low as 8 Gy [33,34]. Additionally, hepatocyte apoptosis due to DNA damage has been documented in all animals exposed to 4 Gy of γ-irradiation [35]. In our study, despite administering a significantly higher dose of electron irradiation (30 Gy), the biochemical and histopathological alterations (hepatocyte swelling, degeneration, and small hemorrhages) were comparable to those observed with lower doses (<10 Gy) of X-ray and γ-irradiation [11]. This finding suggests that electron irradiation presents milder after-effects than other types of ionizing radiation, further supporting its potential as a safer alternative in clinical oncology.

The molecular mechanisms underlying RILD, especially with X- and γ-radiation, have been well-documented [1,36,37]. These can be divided into direct and indirect damage pathways. Direct damage includes the accumulation of unrepaired DNA breaks, leading to miR-34a activation, which halts the cell cycle in the G1 phase and promotes apoptosis through pro-inflammatory cytokines like TNF-α, IL-1, IL-6, and TGF-β [38,39]. These cytokines exacerbate tissue damage by suppressing antioxidant enzymes such as glutathione peroxidase, catalase (CAT), and superoxide dismutase (SOD) [40]. In our study, a significant decrease in SOD levels was observed following 30 Gy of electron irradiation, although this decrease was less pronounced than that typically seen with X- and γ-radiation, indicating a relatively milder oxidative stress response with electron irradiation.

**Figure 11:**
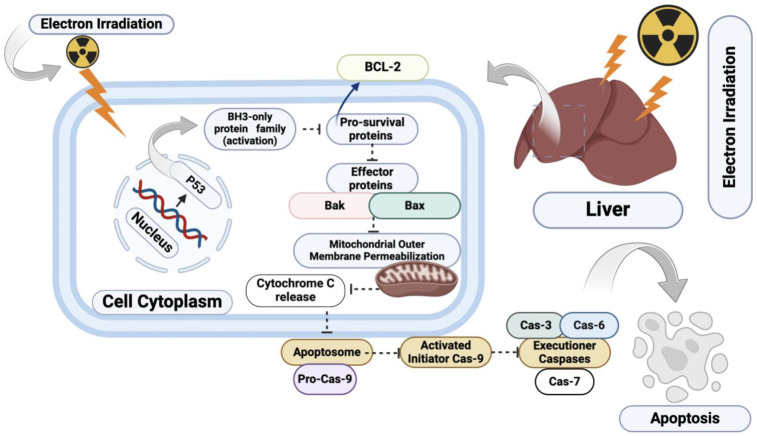
Bax/Bcl-2 pathways in apoptosis activation following electron irradiation. The intrinsic pathway of apoptosis, regulated by the BCL-2 protein family, is triggered by cellular stress, such as DNA damage. The BH3-only protein family (e.g., PUMA, BIM) are upregulated and bind to pro-survival BCL-2 proteins, freeing the pro-apoptotic proteins BAX and BAK. This leads to mitochondrial outer membrane permeabilization (MOMP), releasing cytochrome c into the cytoplasm. Cytochrome c then promotes the formation of the apoptosome, initiating the caspase cascade and leading to programmed cell death.

A crucial role in initiating the intrinsic apoptosis pathway through the Bcl-2 family of proteins is played by p53. It initiates an increase in the expression of the pro-apoptotic proteins Bax and Bak, and a reduction in anti-apoptotic factors like Bcl-2 and Bcl-xl (Figure 11) [41]. These changes enhance mitochondrial membrane permeability, activating caspase 9 and subsequently caspase 3, the final executioner of apoptosis [42,43]. Consistent with this mechanism, our real-time PCR analysis showed significant upregulation of Bax mRNA and elevated expression of caspase-3, indicating a strong apoptotic shift in the liver cells post-irradiation. This was further confirmed by the immunohistochemical examination, which revealed increased caspase-3 immunostaining and reduced proliferative activity, as evidenced by lower Ki-67 values.

**Figure 12:**
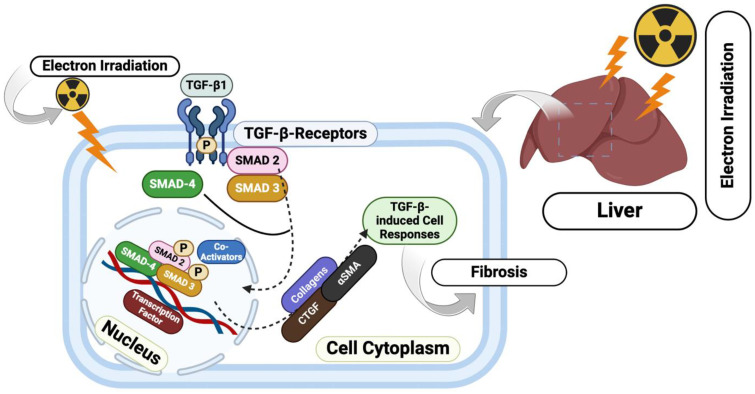
Mechanisms of fibrosis formation induced by electron irradiation in liver tissue and activation of HSCs. The figure illustrates the TGF-β signaling pathway, where TGF-β binds to its receptors, leading to the phosphorylation and activation of SMAD proteins. These activated SMADs form complexes that translocate to the nucleus, where they regulate gene expression, driving fibrogenesis and other cellular responses.

The presence of evidence of moderate fibrotic changes 3 months after electron irradiation is attributed to the inflammatory response in the early period of RILD, and possibly due to the activation of hepatic stellate cells (HSCs), which play a key role in the pathogenesis of radiation-induced liver fibrosis [44,45,46] through the production of TGF-β1 and the subsequent activation of TGF-β1/Smad/CTGF (Figure 12).

Vitamin C, a potent antioxidant and free radical scavenger [47,48], was selected as a radioprotector in this study due to its dual ability to mitigate both indirect and direct radiation damage. This substance not only neutralizes reactive oxygen species (ROS), reactive nitrogen species (RNS), and lipid peroxidation products across various organs during irradiation but also exhibits a unique capacity to protect DNA from electron-induced damage by reducing Cu^2+^ ions, which was shown in in vitro studies [49,50]. Among antioxidants, vitamin C stands out for its ability to reduce single- and double-strand DNA breaks, thereby offering robust protection against radiation damage.

In our study, we used a dose of 50 mg/kg, which was found to be optimal, balancing high radioprotective activity with a low risk of adverse effects. This dosage effectively minimized radiation-induced liver damage (RILD) at multiple levels of biological organization, from the molecular to organ system levels. Pre-irradiation administration of vitamin C significantly improved the morphological condition of the liver, helped maintain the proliferation–apoptosis balance, and modulated cytokine expression, thereby demonstrating both anti-apoptotic and anti-inflammatory effects.

To further evaluate the molecular effects of electron beam irradiation and vitamin C administration, real-time PCR was conducted to assess the expression levels of the Bax, Bcl-2, and caspase-3 genes in liver tissues. The results indicated significant alterations in gene expression across the experimental groups, consistent with the expected apoptotic and anti-apoptotic responses. In the irradiated group, a substantial upregulation of Bax mRNA expression was observed, indicating increased pro-apoptotic signaling. This was accompanied by an elevation in caspase-3 mRNA levels, correlating with enhanced apoptotic activity. Conversely, the expression of the anti-apoptotic gene Bcl-2 was also elevated but to a lesser extent, resulting in an increased Bax/Bcl-2 ratio that suggested a tilt towards apoptosis.

**Figure 13:**
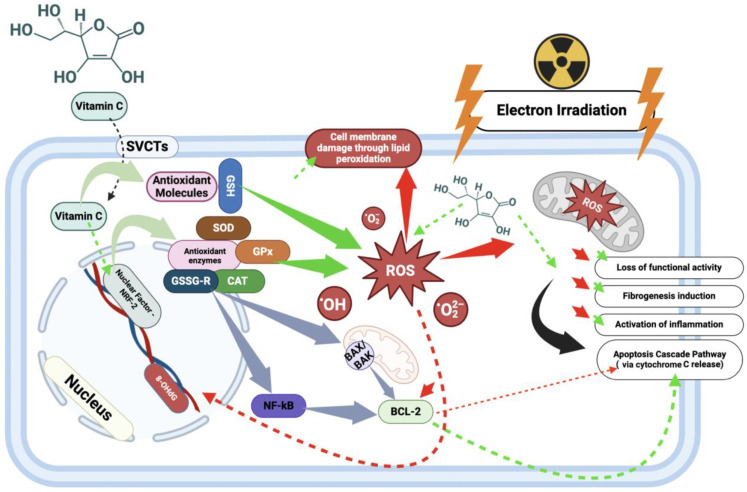
Antioxidant role of vitamin C in protecting liver cells from radiation-induced oxidative stress. The figure illustrates the protective mechanisms of vitamin C against radiation-induced oxidative stress in liver cells. Vitamin C, acting as an antioxidant, neutralizes reactive oxygen species (ROS), thereby reducing their harmful effects on DNA, lipids, and proteins. By stimulating antioxidant enzymes and regulating signaling pathways such as the NRF-2 and other pathways, vitamin C helps maintain cellular homeostasis and reduces apoptosis, ultimately preventing fibrosis and inflammation in liver tissue.

The observed reduction in apoptotic hepatocyte death likely stems from decreased organelle damage (in Group IV), which shifted the cellular response towards reparative mechanisms rather than apoptosis. Vitamin C was shown to induce endogenous antioxidant defense enzymes, including superoxide dismutase (SOD), while reducing oxidative stress markers like malondialdehyde (MDA). It also likely enhances the activity of other antioxidant enzymes such as glutathione-S-transferase, glutathione peroxidase, and catalase. On the molecular level, vitamin C directly binds and neutralizes ROS (O_2_•^−^, OH•, and H_2_O_2_) and RNS (NO• and ONOO^−^), restoring redox homeostasis, potentiating the effects of endogenous low-molecular-weight antioxidants like GSH, tocopherol, and coenzyme Q, and inducing transcription factors for antioxidant enzymes (Figure 13) [51]. Furthermore, vitamin C exhibited a pronounced anti-inflammatory effect by suppressing the expression of pro-inflammatory cytokines (IL-1β, IL-6, and TNF-α), aligning with the findings of other studies.

Thus, the present study is the first to demonstrate the protective effects of vitamin C pre-administration in the context of electron beam irradiation of the liver. The results revealed significant modulation of apoptotic pathways, as evidenced by alterations in the expression of Bax, Bcl-2, and caspase-3. However, the precise molecular mechanisms underlying these protective effects remain unclear and warrant further investigation. It is likely that vitamin C’s ability to attenuate oxidative stress and influence apoptotic signaling plays a crucial role in reducing radiation-induced liver damage. This novel finding suggests that vitamin C may serve as an effective radioprotector, but additional studies are needed to fully elucidate its mechanism of action and to explore its potential in clinical applications for the prevention of radiation-induced liver disease (RILD). Further research could also aid in the development of targeted therapies for mitigating radiation-induced liver damage in cancer patients.

## 5. Conclusions

Our study presents the first evidence of vitamin C’s radioprotective effects in a model of electron beam-induced liver damage. Vitamin C exhibited substantial antioxidant, anti-apoptotic, and anti-inflammatory properties during both the acute and long-term phases following irradiation. Notably, its administration mitigated the progression of radiation-induced liver fibrosis, likely through the modulation of key apoptotic signaling pathways, such as the Bax/Bcl-2 axis, and the reduction in oxidative stress markers, including superoxide dismutase (SOD) and malondialdehyde (MDA). These findings highlight vitamin C’s potential as an effective radioprotector against electron irradiation-induced liver damage. Furthermore, these findings open new horizons for the incorporation of vitamin C into radiotherapy protocols, particularly in outpatient settings, with the potential to significantly enhance the quality of life for patients undergoing radiotherapy. Nevertheless, further research is essential to fully elucidate the precise molecular mechanisms involved and to explore the broader clinical implications of these protective effects.

## Figures and Tables

**Table 1:** Bax, Bcl-2, caspase-3 gene information.

Gene	Primer Sequences	Melting Temperature of Primers (°C)	Product Size (bp)	References
**Bax**	F—ATGGAGCTGCAGAGGATGATT R—TGAAGTTGCCATCAGCAAACA	60	97	[25,26]
**Bcl-2**	F—TGGGATGCCTTTGTGGAACT R—TCTTCAGAGACTGCCAGGAGAAA	60	73	[25,27]
**Caspase-3**	F—AATTCAAGGGACGGGTCATG R—GCTTGTGCGCGTACAGTTTC	60	67	[28,29]

## Data Availability

The study did not generate publicly available archival data.
